# Transcriptional Response Of *E. coli* Upon FimH-mediated Fimbrial Adhesion

**DOI:** 10.4137/grsb.s4525

**Published:** 2010-03-24

**Authors:** Prasanna Bhomkar, Wayne Materi, Valentyna Semenchenko, David S. Wishart

**Affiliations:** 1 Departments of Biological Sciences and Computing Science, University of Alberta, Edmonton, Alberta, Canada T6G 2E8; 2 National Research Council, National Institute for Nanotechnology (NINT), Edmonton, Alberta, Canada T6G 2M9. Email: bhomkar@ualberta.ca

**Keywords:** adhesion, biofilm, bacteria, fimbriae, microarray

## Abstract

Functionalities which may be genetically programmed into a bacterium are limited by its range of possible activities and its sensory capabilities. Therefore, enhancing the bacterial sensory repertoire is a crucial step for expanded utility in potential biomedical, industrial or environmental applications. Using microarray and qRT-PCR analyses, we have investigated transcription in *E. coli* (strain CSH50) following FimH-mediated adhesion to biocompatible substrates. Specifically, wild-type FimH-mediated adhesion of *E. coli* to mannose agarose beads and His-tagged FimH-mediated adhesion to Ni^2+^-NTA beads both led to induction of *ahpCF*, *dps*, *grxA* and *marRAB* genes among bound cells relative to unbound cells. The strongly-induced genes are known to be regulated by OxyR or SoxS cytoplasmic redox sensors. Some differentially altered genes also overlapped with those implicated in biofilm formation. This study provides an insight into transcriptional events following FimH-mediated adhesion and may provide a platform for elucidation of the signaling circuit necessary for engineering a synthetic attachment response in *E. coli*.

## Introduction

Programming complex behavior in bacteria requires the construction of synthetic sensory, logic and effector circuits.^1^–^3^ The ability of genetic logic circuitry to compute interesting and useful behaviors is limited by the sensory repertoire of the cell, which historically has been restricted to molecular sensors such as those for nutrients, nutrient analogs, metals and selected small molecules. Noticeably missing from this list is the bacterial equivalent of an “attachment” sensor that might be activated upon binding to a recognizable substrate.

Fimbriae are long, thread-like surface structures present on several bacteria which enable them to adhere to and colonize specific host tissues. Many gram negative bacteria, including a majority of *E. coli* strains may express up to 300–500 copies of Type 1 fimbriae on their outer membranes enabling them to bind to mannosylated residues of bladder or intestinal epithelial cell surface proteins. Measuring up to 2 μm in length and consisting of a 7–10 nm diameter rod, Type 1 fimbriae are mainly composed of repeating sub-units of FimA protein, capped by a 3 nm diameter distal protein complex composed of FimF, FimG and the mannose-recognizing FimH protein.^4,5^ Mechanical stress, such as that imposed during fluid flow, stimulates stronger binding between FimH and its cognate ligand due to formation of “catch bonds”.^6,7^

A substantial amount of literature suggests that sensing systems are activated when individual *E. coli* cells come into contact with surfaces and form biofilms. For instance, random transposon mutagenesis of an *E. coli* K12 mutant strain able to colonize hydrophobic (glass) and hydrophilic (polystyrene) materials revealed 98 genes that were significantly up-regulated in attached cells and 73 with reduced expression after 24-hours of colonization, notably including members of the two-component Cpx signaling system.^8,9^ The interaction of *E. coli* with abiotic surfaces via P-pili triggers the Cpx pathway, the signal transduction of which is dependent on the outer membrane protein NlpE, presumed to be the direct sensor of contact with a surface.^10^ Yet, little is known about the *E. coli* response upon attachment to bio-compatible surfaces and much less is understood about the responsible sensory signal transduction mechanism. An important goal of our study was, therefore, to ascertain whether shear stress imposed on *E. coli* attached to a mannosylated substrate via FimH-mediated fimbrial adhesion resulted in a detectable transcriptional response.

Differential display analysis of the related PapG-mediated fimbrial adhesion of a uropathogenic *E. coli* strain to erythrocyte surface glycoproteins has previously been shown to activate *barA* transcription.^11^ A more recent study^12^ also employed differential display PCR to examine the response of FimH-mediated Type 1 fimbrial binding of a separate uropathogenic *E. coli* strain to a more well-defined surface (mannose-sepharose beads) and identified the capsular assembly gene *kpsD* to be down-regulated upon attachment.

We have used both microarray analysis and quantitative RT-PCR (qRT-PCR) to analyse the *E. coli* transcriptome during the early stages of FimH-mediated Type 1 fimbrial adhesion to appropriately functionalized agarose beads. By utilizing agarose beads in a manner similar to that done previously,^12^ we have been able to focus on an important part of the adhesion response. An analysis of affected genes identified several which are known to be regulated by either OxyR or SoxRS sensors of cellular redox status. However, transcription of other genes with unknown activators was also observed, suggesting that multiple sensory and response pathways may be involved. We also followed the transcript profile after four and eight hours of fimbrial adhesion and observed larger increases or decreases in expression levels with some responsive genes, while others returned to normal levels over time.

Earlier studies pertaining to fimbrial-mediated *E. coli* adhesion dealt with pathogenic strains which makes them unsuitable for our objective of designing and manipulating bacteria as biological devices to fulfill important goals in synthetic biology.^11,12^ In this study, we have used a benign laboratory strain of *E. coli*, namely CSH50, which binds to mannose via the FimH adhesin, as a model for our investigation. Molecular characterization of the *E. coli* FimH using directed and random mutagenesis has previously identified sites outside of the lectin domain into which heterologous sequences could be inserted without compromising FimH functionality.^13,14^ In this study, we were also interested to engineer a histidine-tagged version of FimH that would alter the normal mannose-specificity in favor of nickel binding. We compared the transcriptional responses upon binding to nickel-based versus mannose-based substrates for a subset of consistently upregulated genes and found similar responses for both substrates, suggesting the regulatory components of these genes might be candidate transcriptional reporters for an attachment response. We believe that our results offer a glimpse of early transcriptional output following FimH-mediated fimbrial adhesion and open possible avenues to engineer “attachment” mediated responses in *E. coli*.

## Experimental Procedures

### Bacterial strain and growth conditions

Expression of the *fim* operon (*fimAICDFGH*) is controlled by orientation of the promoter (pA) present in an invertible switch element (*fimS*) located immediately upstream of *fimA*. Two recombinases, FimE and FimB, catalyze the inversion of the switch resulting in either an “ON to OFF” or “OFF to ON” orientation respectively. The strain CSH50 (*fimE1::IS1-, rpsL-(strR), araBAD-0, thi-,DE(pro-lac)*), obtained from The Coli Genetic Stock Center, Yale University, is deleted for the FimE recombinase resulting in a hyper-fimbriated phenotype. Cells were grown in LB medium supplemented with 35 μg/ml streptomycin at 37 °C and shaken below 50 rpm in order to prevent fimbriae from being sheared off during growth. CSH50 expressing the histidine-tagged version of FimH was grown in LB supplemented with 35 μg/ml streptomycin and 50 μg/ml ampicillin under same conditions as mentioned before.

### Small scale binding assay and microscopy

Approximately 250 μl of cells from a mid-log phase culture was added to 40 μl of mannose agarose beads (Sigma Cat. no. M6400) in a 1.5 ml microfuge tube and these tubes containing the bacterial culture-bead suspension were rotated at 360 degrees for 30–40 minutes at 25 °C using a Barnstead Labquake tube rotator (Thermo Scientific Inc.). The microtubes were allowed to stand for a minute (to let the beads settle under gravity) followed by removal of the supernatant (consisting of cells that were either unattached or loosely attached to the beads). The settled beads were washed twice with 1 ml LB before resuspending in 200 μl LB. Forty microlitres of the bead suspension was transferred onto a slide and visualized by phase contrast microscopy at 20 X magnification (Olympus IX81 inverted microscope). Images were collected using Image Pro Plus software (Media Cybernetics). Preliminary experiments revealed that pretreatment of CSH50 cells with 10–20 mM D-mannose was sufficient to block the FimH-mediated binding to mannose agarose beads. Also, addition of mannose to CSH50 cells attached to mannose-functionalized agarose beads resulted in detachment of the bound cells from the beads. These two experiments clearly confirm that the CSH50 cells were specifically attached via the FimH adhesin to the mannose moiety of the agarose beads.

### Adhesion assay

Mannose agarose beads were used to bind CSH50 cells while Ni^2+^-NTA agarose beads (Qiagen Cat. no. 30210) were used as the corresponding negative control. Large-scale adhesion assays were performed in disposable Econo-Pac chromatography columns (BioRad, Cat. no. 732–1010; 1.5 × 12 cm). These columns are pre-fitted with a porous 30 μm filter that helps retain the beads and any attached cells, while allowing unattached cells to flow through. Approximately 8 ml bead slurry was poured into a column to give a settled bead volume of 4 ml.

Cell cultures for each assay were initiated by inoculating 50 ml of LB plus appropriate antibiotic with 1 μl of a saturated CSH50 culture and grown overnight at 37 °C with 50 rpm to an A_600_ of 0.4 prior to being added to columns containing beads. Specifically, 10 ml aliquots from the same culture were added either to a column containing mannose agarose beads (the bound condition) or to a column containing Ni^2+^-NTA agarose beads (the unbound condition) and incubated at room temperature (approximately 22 °C) with gentle rotation for one, four or eight hours. Following incubation, the column containing mannose agarose beads was drained of LB medium under gravity to remove unattached cells prior to elution of the bound cells. The unbound cell fraction was eluted from the other column (with Ni^2+^-NTA agarose beads) by draining. In order to elute cells bound to the mannose-agarose beads, 5 ml of plain LB medium was added to the column, vortexed for 30 sec and drained. Eluted cells from each condition were immediately collected in 1.25 ml of cold stop solution (5% water-saturated phenol, pH 7.0, in ethanol) for RNA isolation.

CSH50(WMHis6) cells were treated similarly except that they were incubated with Ni^2+^-NTA agarose beads (bound condition) and Ni^2+^-NTA agarose beads treated with NaOH-EDTA solution to remove coordinated Ni^2+^ (unbound condition). Cells bound to Ni^2+^-NTA agarose beads were detached by addition of 5 ml LB medium supplemented with 500 mM imidazole, following which the cells were immediately eluted. The corresponding unbound cell fraction was obtained by simply draining the column with stripped NTA agarose under gravity. Eluted cells were immediately treated with cold stop solution as above and RNA was extracted as below.

### RNA extraction

A three ml aliquot of eluted cells was centrifuged at 13,000 rpm for 1 min and the supernatant was discarded. RNA was extracted using the MasterPure RNA purification kit MCR 85102 (Epicentre Technologies, Madison, WI) according to the manufacturer’s instructions, with slight modifications to optimize yield and purity. Prior to treating with DNaseI, total nucleic acid was quantified by spectrophotometry on a NanoDrop ND-100 (NanoDrop Technologies Inc. Wilmington, USA) and 20 μg of nucleic acid from each sample was treated with 20U of DNaseI for one hour. RNA integrity was assessed using the Agilent 2100 Bioanalyzer (Agilent Technologies, CA). The A_260/280_ for all RNA samples was in the 2.0–2.15 range while the 23S/16S RNA ratios were between 1.2 and 1.4 with RNA integrity numbers (RINs) between 7.8 and 8.3. RNA from all samples was diluted to 500 ng/μl before being used for microarray or quantitative real time PCR.

### Microarray analysis

Custom microarrays were printed at the University of Alberta Molecular Biology Services Unit (MBSU) microarray facility using Corning Epoxide slides (for more information, see http://www.biology.ualberta.ca/facilities/mbsu/). The Operon *E. coli* Genome Oligo Set version 1.0 (Operon Biotechnologies, Inc.) is an anti-sense oligo set consisting of 70-mer probes representing 5,978 ORFs derived from three strains of *E. coli* along with additional positive and negative controls. Each microarray contains three complete duplicates in separate blocks.

A Genisphere Array 900MPX Expression Array Detection Kit was used to produce Cy3 or Cy5 labelled cDNA as per the manufacturer’s instructions. Briefly cDNA was produced by reverse transcription of 2–3 μg total RNA using random hexamers (included in the kit), then poly T-tailed with terminal deoxy-transferase. A bridge oligo was used to associate and then ligate specific capture sequences to the T-tailed cDNAs. Different bridge oligos are associated with a sequence that is complementary to either Cy3- or Cy5-labelled dendrimers. Thus one of the cDNAs (e.g. for “bound” cells) was ligated to a Cy3-specific capture sequence while the other (e.g. for “unbound” cells) was ligated to a Cy5-specific capture sequence. The two cDNA: capture sequence fusions were mixed, placed on a BSA-blocked microarray, covered with a 54 mm LifterSlip (Erie Scientific Company, Portsmouth, USA) and hybridized overnight at 60 °C in a humidified chamber. Separately, cDNAs with dye-swapped capture sequences were also prepared and similarly hybridized to another microarray.

The next day the LifterSlip cover slip was gently removed in 2X SSC, 2% SDS and successively washed for 5 min. in 2X SSC, 2% SDS then 2X SSC and finally 0.2X SSC by serial transfers in disposable Coplin jars. Slides were dried by placing label-side down in a disposable Coplin jar with an absorbent disc in the bottom and spin-dried at 3000 rpm for 3 min. A Cy3/Cy5 dendrimer mixture was then hybridized to each array for 4 h at 60 °C followed by washes and spin-drying as above. Microarrays were scanned at 5 μm/pixel resolution in an ArrayWorX biochip reader (Applied Precision) using GenePix 6.0 (Molecular Devices Corporation). Independent microarrays conducted after different time intervals using the original RNA and RNA samples extracted from independent biological replicates gave us similar results indicating that microarrays and the binding assays were performed under reasonably reproducible conditions.

### Statistical analysis of microarray data

Separate GAL files were used to process each repeat block on a microarray. This gave a maximum of 12 data points for each gene (2 experiments X 2 arrays for each dye orientation X 3 replicates per array). Negative control (empty, buffer only or random negative control oligos) and poor-quality spots were filtered from the data and result files were transferred to Excel (Microsoft Inc.). The data were scaled so that the average Median of the Ratios for the total spots in a single replicate block was 1.000. A two-tailed, unequal variance Student’s t-test was performed comparing the distribution of the data points for a single gene to that of all genes. The data set was then sorted in order of ascending *P*-value and only genes that were up- or down-regulated more than 1.5-fold with a *P*-value less than 0.010 were considered for subsequent analysis.

### Quantitative RT-PCR

cDNA was synthesized from 4 μg total RNA using Superscript III and random hexamer primers (Invitrogen). Gene sequences were obtained from the Regulon DB V5.7 database (http://regulondb.ccg.unam.mx/index.html) and were used to design qRT-PCR primers (amplicon size 70–80 bp) using Primer Express 2.0 (Applied Biosystems, USA). Primers were purchased from Integrated DNA Technologies, Canada. The genes that were validated as well as the primers used in these experiments are listed in Table 3. The qRT-PCR was performed with 1X SYBR Green master mix [Tris (pH 8.3), KCl 50 mM, MgCl_2_ 3 mM, 0.8% glycerol, 0.01% Tween 20, 2% DMSO, 0.2 mM dNTPs, ROX (passive reference dye), SYBR Green, 0.03 units/μl Platinum Taq] in a 10 μl reaction volume. The reactions were carried out in 96-well plates using the ABI 7500 detection system (Applied Biosystems) according to the manufacturer’s instructions. Each reaction comprised 2.5 μl cDNA, 2.5 μl of primer (1.6 μM each) and 5 μl of 2X SYBR Green master mix. The initial denaturation time was 2 min at 95 °C, followed by 35 cycles of 95 °C for 15 sec, 60 °C for 1 min. Following PCR amplification, a dissociation curve was run to examine the amplification specificity. A portion of the cDNA was diluted to 1/4, 1/16, 1/64, 1/256, 1/1024 and 1/4096 and 2.5 μl of each dilution was used for primer validation and determination of optimal template dilutions. Relative expression levels were estimated using the comparative critical threshold (ΔΔCt) method (RTL = 2 ^(ΔCt sample – ΔCt reference condition)^).^15^ The gene (*yqaB*) was used as an internal control because it remained constant under all conditions tested (data not shown). qRT-PCR for each gene was performed in triplicate for each of the three biological replicates.

### Construction of histidine tagged versions of FimH

**Figure 1.**
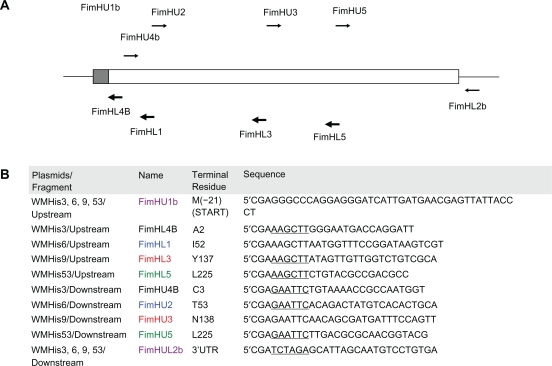
Cloning histidine-tagged *fimH*. **A**) Location of primers used for inserting histidine-tag(s) at different positions in the mature FimH protein. The shaded grey box represents the signal peptide that is cleaved off during maturation of the FimH protein. Thick arrows represent primers used for amplifying the upstream fragment and thin arrrows represent those used to amplify the downstream fragment **B**) Primers used for *fimH* amplification (underlined bases represent appropriate restriction sites while bolded sequence represents the canonical Ribosome Binding Site i.e. RBS).

pBSDe1Z is pBluescript KS(+) with the 18 N-terminal residues (from the start of LacZ translation to the *KpnI* site) removed by long inverse PCR. It was constructed so that new coding regions could be inserted within the multi-cloning site (MCS) without resulting in LacZ translational fusions. In order to effect binding of modified FimH protein to immobilized nickel (Ni^2+^-NTA agarose beads), we selected four different positions (WMHis3 = A2, WMHis6 = I52, WMHis9 = Y137 and WMHis53 = Q224) after which to insert six (His) or twelve (2His) histidine residues. The I52 and Y137 residues of the mature FimH have previously been shown to interact with the cognate ligand,^16,17^ while histidine-tag inserts near Q224 (i.e. A225) have been shown previously to allow FimH-mediated binding to Ni^2+^-NTA agarose beads without interfering with mannose binding of the recombinant FimH.^18,19^ Fragments from *fimH* were prepared by long and accurate PCR using *E. coli* genomic DNA as template, adding appropriate restriction sites to the respective ends of the resulting fragments for cloning purposes (Fig. 1).

Each plasmid was constructed by first cloning the “upstream” fragment into pBSDelZ between the *ApaI* and *HindIII* sites of pBSDelZ, followed by insertion of the His tag (between the *HindIII* and *EcoRI* sites) and finally the insertion of the “downstream” fragment between the *EcoRI* and *XbaI* sites in the nascent plasmid. Each cloning step was verified by sequencing. The upstream fragment was prepared using an “upstream” primer pair (FimHU1b and a fragment-specific primer) which added an *ApaI* restriction site and canonical Ribosome Binding Site i.e. RBS (5′AGGAGG) at the 5′ end of the fragment and a *HindIII* site at the 3′ end. The downstream fragment was prepared using a “downstream” primer pair (FimHL2b and a fragment-specific primer) which added an *EcoRI* site at the 5′end of the fragment and an *XbaI* site at the 3′ end. Finally the 6-His insert was prepared by annealing 5′ phosphorylated oligonucleotides HisHEUP (5′AGCTTCATCATCATCATCATCATG) and HisHELP (5′AATTCATGATGATGATGATGATGA), resulting in 5′ *HindIII* and 3′ *EcoRI* cohesive sites (underlined). The addition of a 5′ *HindIII* site added an KL residue pair amino to the 6-His insert while the 3′ *EcoRI* site addsed a carboxyl EF residue pair to the insert. In addition, we created a 12-His insert to insert at Q224 using annealed 5′ phosphorylated oligos His12HEU (5′AGCTTCATCACCATCATCACCATAGATCCCATCACCATCATCACCATG) and His12HEL (5′AATTCATGGTGATGATGGTGATGGGATCTATGGTGATGATGGTGATGA). This insert added a total of 18 residues (KLHHHHHHRSHHHHHHEF) after Q224 in the mature protein.

Constructs were verified by sequencing, before mobilizing them into CSH50 chemically competent host cells. Single colonies were picked to initiate cultures in LB medium and 200 μl of early log phase cultures (OD_600_ = 0.3) tested for their ability to bind Ni^2+^-NTA agarose beads. Glycerol stocks were made from cultures verified to be able to bind to the beads and were used for subsequent microarray and qRT-PCR experiments.

## Results and Discussion

The desire to identify the response mechanism used by *E. coli* to signal that it is attached to a natural biological substrate led us to investigate the transcriptome following FimH-mediated fimbrial binding of cells to mannose-agarose beads. A relatively short binding period (one to eight hours) was employed compared to longer intervals (one to eight days) used in previous studies that explored transcriptional changes during biofilm formation. Congruent with our vision in synthetic biology, we were also interested in investigating whether the transcriptional response of binding to nonnative target substrates, using a genetically modified FimH, would trigger events similar to those elicited by binding to mannose. We therefore made directed changes in the FimH adhesin aimed at enabling the transgenic cells to attach to Ni^2+^-NTA agarose and investigated the resultant transcriptomic response. We hope that results obtained from the present study will lay the groundwork to eventually decipher the relevant sensory mechanism following FimH-mediated fimbrial adhesion and enable the construction of cellular modules responsive to attachment.

### Microarray analysis of the transcriptional response to fimbrial-mediated adhesion

A single CSH50 mid-log phase culture was subdivided into two aliquots. One aliquot was incubated with LB-equilibrated mannose-agarose beads for one hour (bound cell fraction) while the other aliquot was similarly incubated in an equal volume of LB-equilibrated Ni^2+^-NTA agarose beads (unbound cell fraction). Comparative microarrays were prepared from concentration-normalized RNA extracted from the bound and unbound cell fraction. Following filtering of negative control points and poor quality spots, median of ratio data within each replicate were normalized to a mean of 1.0 and a two-tailed Student’s t-test was calculated comparing data for each gene to the entire microarray set.

At a 0.01 level of significance and a 1.5 fold cut-off for change in transcript ratio, 42 genes were found to be differentially expressed (Table 1). Twelve genes were up-regulated by 2-fold or more while, of the remaining 30 affected genes, 22 were up-regulated by 1.5—less than 2 fold and the rest were down-regulated. All the members of three operons (*ygaVP*, *emrRAB* and *marRAB*) were represented in the up-regulated gene set. Gene functions were annotated according to the EcoCyc and CyberCell databases and supplemented by specific literature searches.^20,22^ The majority of these changes were localized to four functional groups, namely protective metabolic pathways, general metabolism, transport/transport-related genes and proteins with hypothetical or unknown functions. We observed an increase in the expression of genes involved in protection of cells from oxidative damage and hydrophobic compounds, mediating export of potential damaging agents, while down-regulated genes predominantly included those involved in nutrient import and general metabolism.

Changes in genes related to protective metabolism were analyzed using the Biocyc Pathway Tools version 11.5 (http://biocyc.org/expression.html). Genes belonging to three pathways (assimilatory sulfate reduction I, glutathione-dependent formaldehyde degradation, and removal of reactive oxygen species) exhibited increased transcription, suggesting that cells increase their anti-oxidant and related activities significantly upon fimbrial adhesion.

A total of five transport-related genes (*emrB*, *marB*, *yeeE*, *glnH*, *yicE*) were also significantly affected by fimbrial-mediated binding (Table 1). Up-regulated transport genes (*emrB*, *marB*, *yeeE*, *glnH*) are mainly involved in export of potential damaging agents such as antibiotics and drugs (though some of these assignments are only putative) while the down-regulated gene, *yicE*, is a putative transporter of unknown function.

#### Redox responsive regulons were affected

**Figure 2.**
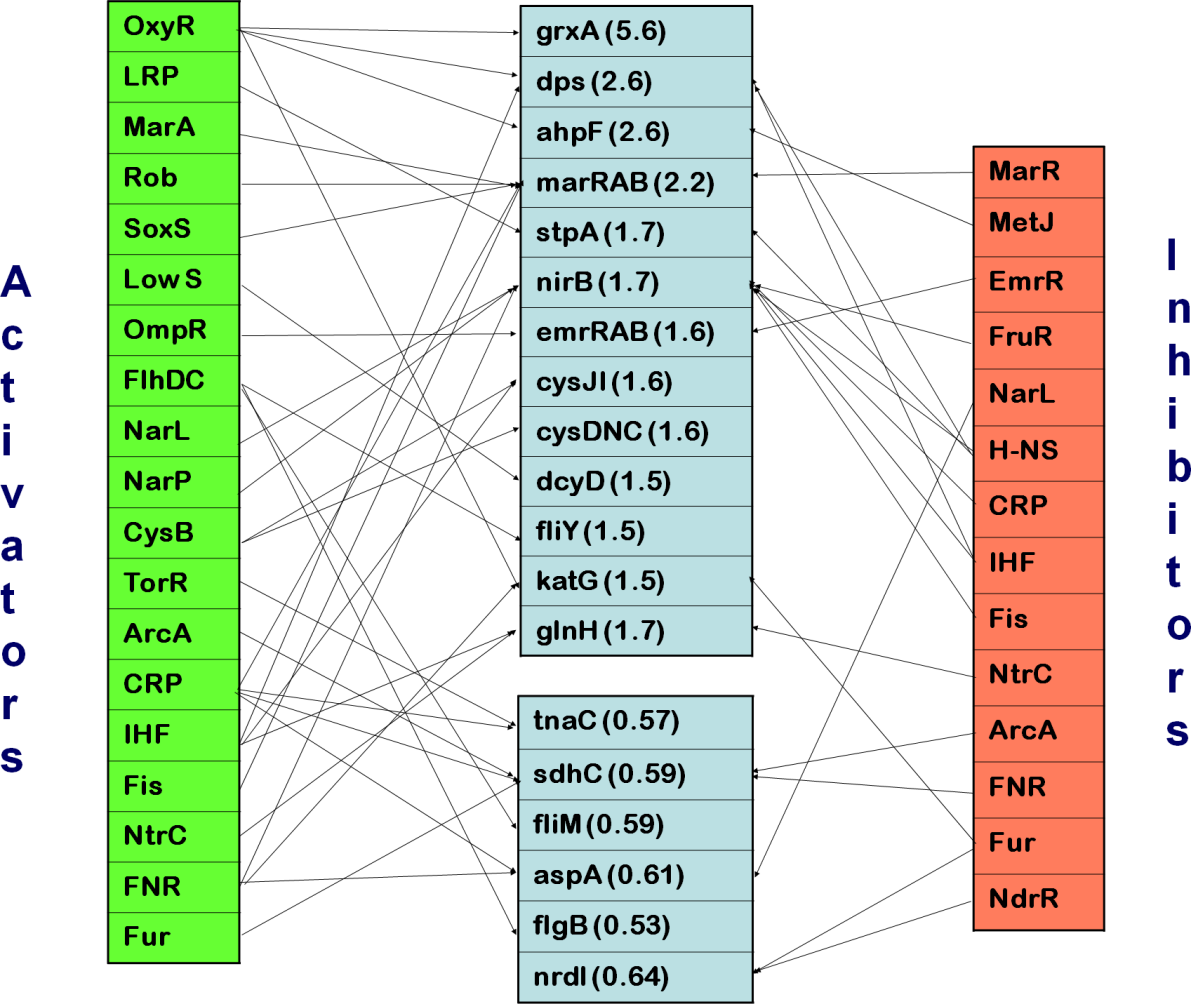
Regulation of genes affected by fimbrial adhesion. Genes with altered transcription levels are shown in center boxes with average fold-increase or decrease (according to microarray analysis following one hour of binding) shown in parentheses. Regulators are connected by arrows to genes which they regulate; activating regulatory factors are shown on the left while inhibiting factors are shown on the right. The differentially altered genes were analyzed using regulatory data from RegulonDB V6.3.

In order to determine the underlying pathways that may be responsible for the changes in gene expression in cells bound to mannose-agarose beads, we analyzed the total set of 42 affected genes using regulatory data from RegulonDB V6.3.^20^ The results are shown in Figure 2. Of the twelve genes that were up-regulated 2-fold or more, the regulation of only six has been studied in detail. Three of these (*grxA*, *dps*, *ahpF*) are activated by the OxyR cysteine-based redox sensor while the other three (*marR*, *marA*, *marB*) are coordinately activated by the SoxS 2Fe-2S-based redox sensor.

The OxyR regulon also includes *katG*, whose expression only increased 1.5-fold in the bound cells at the one-hour interval. Together, the OxyR and SoxS regulated genes represent all the genes with known regulation which were up-regulated by more than 2-fold after one hour of adhesion. Transcription of OxyR and SoxS themselves was only very moderately up-regulated (approximately 1.2-fold) at this interval (data not shown), suggesting that the redox state of these sensory proteins had a larger effect than their concentration.

The regulation of 14 of the remaining 22 up-regulated genes has been well studied so far. Six of these are activated by low levels of cytoplasmic sulfur; *dcyD* directly (possibly by a transcriptional attenuation mechanism), and both *cysDNC* and *cysJI* through the dual transcriptional regulator, CysB, which is activated by low sulfur levels. Protein products of *cysDNC* and *cysJI* are required for assimilatory reduction of sulfate, leading to increased production of the amino acid cysteine, which is used to maintain redox balance through production of glutathione, glutaredoxin and thioredoxin. Four other genes share no common regulators but have a net effect of increasing drug resistance. This includes genes activated by NarL and NarP (*nirB*), and by OmpR (*emrRAB*).

The regulation of 6 of the 8 down-regulated genes has also been well documented. Four of these are regulated by one or more of, CRP, FNR and ArcA, which are all responsive to the cellular redox state. The remaining two down-regulated genes are flagellar components and are governed by the flagellar master regulator, FlhDC.

It was somewhat unexpected that the fimbrial adhesion response seemed to activate or alter genes associated with metabolic activities as opposed to cell surface or cell structure genes. However, tight attachment of cells to a substrate results in their relative immobilization and shields a substantial part of their surface area, likely having some metabolic consequences. Also, a partial loss of cell mobility and the reduction in accessible surface area possibly reduces the cellular capacity to absorb nutrients and oxygen from the media. These events are likely to be the reason that most of the differentially expressed genes were mapped to metabolic pathways which may mediate downstream events following attachment.

### Transcriptional changes verified by qRT-PCR

In order to verify our microarray results, we performed quantitative RT-PCR (qRT-PCR) for selected transcripts. RNA was isolated from bound and unbound cell fractions of three independently performed binding assays and used to amplify cDNA using a set of random hexameric primers that was different from the one used for microarray experiments. The fold-changes for selected transcripts obtained using qRT-PCR were in agreement with those obtained previously with our microarray experiments and the induction ratios derived from qRT-PCR assays generally exhibited higher magnitudes (Table 2).

### Time series analysis of response

In a separate experiment, we also measured the transcriptional responses after one, four or eight hours of fimbrial attachment to mannose-agarose beads using both microarrays and qRT-PCR. Transcriptional responses at each interval were analyzed independently using two microarrays, containing three replicate blocks each and incorporating dye swaps between the arrays. Identical filtering and statistical analysis was carried out as for the one-hour interval. Both qRT-PCR and microarray data reflect similar changes in expression levels of affected genes over these intervals (Table 2). The consistency of the transcriptional data over several time points suggests that we have correctly identified the primary responses. However, after four and eight hours, a number of secondary responses were also engaged and will be analyzed separately (manuscript in preparation).

Genes under the direct control of OxyR (*grxA, dps, ahpF* and *katG*) were increasingly up-regulated as the adhesion time increased. The exception was *dps,* which first underwent a transient decrease after four hours of binding before returning to elevated levels. Expression of SoxS-regulated *marA* was slightly elevated at one hour, but also had a transient reduction at the four-hour interval, before returning to slightly-elevated expression levels at the eight-hour interval. Expression of *ygaP* was initially very high but returned to normal levels within four hours of binding. Taken together, these data indicate that OxyR regulated genes had increased levels of induction with prolonged fimbrial-mediated adhesion while SoxS regulation of *marA* was more complex, perhaps reflecting the known positive and negative feedback.

**Figure 4.**
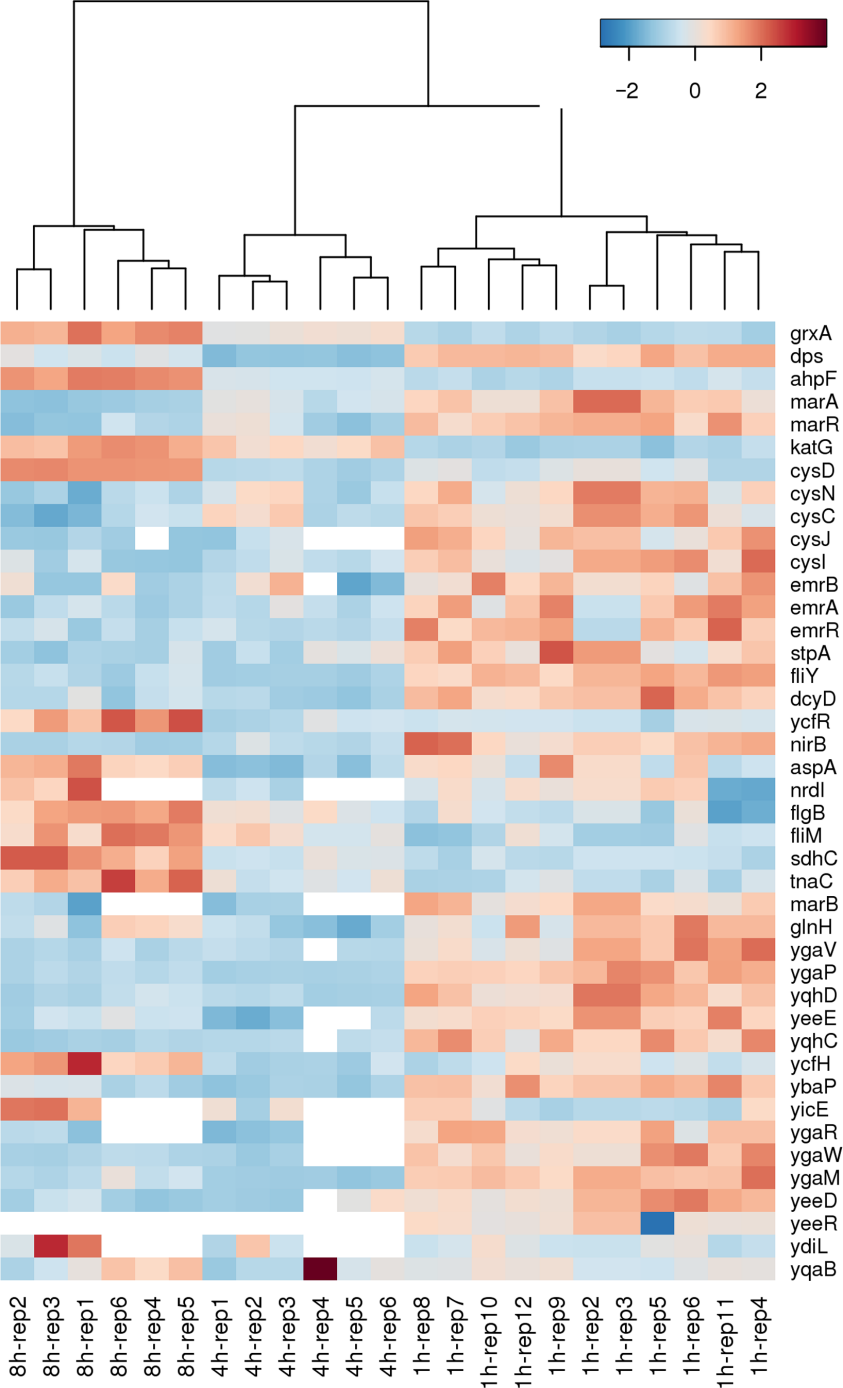
Heat map generated via hierarchical clustering analysis showing differentially altered genes in a temporal manner during *E. coli* attachment. These genes were organized using a hierarchical clustering algorithm (Metabominer; Wishartlab) so that those which display similar expression patterns were grouped together. The hierarchical cluster analysis was performed using average agglomeration method. The heatmap was plotted with gene (row) normalized and the distance between genes were calculated based on Euclidean distance. A color bar is represented at the top of the panel with a range from −2 to 2 (blue to red), with red color representating up-regulation while blue color representing down-regulation.

Comprehensive DNA microarray studies have been previously performed to generate an insight into processes accompanying *E. coli* attachment to surfaces. However, most of them have captured single-time point snap-shots of the transcriptome after relatively long intervals upon attachment to abiotic surfaces, primarily glass, for 32 hours,^23^ 5 to 8 days,^24^ 7 hours,^25^ 9 hours,^26^ 42 hours.^27^ A temporal analysis of the transcriptome over shorter periods of biofilm formation on glass (4, 7, 12 and 24 h) has, however, been recently undertaken.^28^ To our knowledge, our study is one of the first to have employed earlier time points (1 h, 4 h and 8 h) in monitoring transcriptional changes associated specifically with FimH-mediated adhesion of *E. coli* to biotic surfaces. A heat map analysis was performed on the microarray data of 42 genes differentially altered during *E. coli* attachment to mannose agarose beads in a temporal manner. This study revealed that a majority of these genes were induced in the first hour following cell attachment to the substrate and displayed reduced expression levels after four hours (Fig. 4). However, a small subset of genes (namely *grxA, ahpF, katG, cysD, ycfR, tnaC and ycfH*) showed higher levels of transcription upon prolonged attachment periods.

### CSH50 cells with His-tagged FimH bind robustly and specifically to Ni^2+^-NTA agarose beads

The X-ray crystal structure of the FimH-FimC complex, elucidated first by Choudhury et al [1999] and later corroborated by Hung et al^17^ provided definitive evidence of a carbohydrate-binding pocket at the FimH lectin domain. Specifically, residues N46, D47, D54, Q133, N135, D140 and the NH2-terminal amino group of FimH are hydrogen bonded to D-mannose, while I13, Y48, I52 and F142 form a hydrophobic ridge in close association with the mannose-binding pocket.

Based on these studies, we constructed two histidine-tagged versions of FimH, namely WMHis3 (with a hexa-histidine tag inserted after A2 near the amino-terminal) and WMHis53 (with a hexa-histidine tag inserted after the permissive position Q224 near the C-terminal). However, CSH50 cells transformed with either WMHis3 or WMHis53 were unable to bind to Ni^2+^-NTA agarose beads. We therefore constructed three additional tagged versions of FimH, namely WM2XHis3 (which had two hexa-histidine tags following A2), WM2XHis53 (which had two hexa-histidine tags following Q224) and WMHis6 (wherein a single histidine tag was inserted following residue I52). We observed that CSH50 cells transformed with WM2XHis3, WM2XHis53 and WMHis6 bound to Ni^2+^-NTA agarose beads, although fewer CSH50(WM2XHis3) and CSH50(WM2XHis53) cells were found to bind as compared to CSH50(WMHis6) cells (Fig. 3a). An average of only 18 cells of CSH50(WM2XHis53) or 55 cells of CSH50(WM2XHis3) bound to every Ni^2+^-NTA agarose bead. On the other hand, 325 cells of CSH50(WMHis6) bound to every Ni^2+^-NTA agarose bead which was very close to the positive control treatment (CSH50 cells incubated with mannose-agarose beads), wherein 422 CSH50 cells were found to attach to every mannose-agarose bead (Fig. 3b).

To demonstrate that the binding of CSH50(WMHis6) expressing a nickel-binding version of FimH was specific, we pretreated these cells with 100 mM free histidine for one hour before incubating them with Ni^2+^-NTA agarose beads. Binding of histidine-treated cells to Ni^2+^-NTA agarose beads was abolished. In addition, CSH50(WMHis6) cells bound to Ni^2+^-NTA agarose beads could be displaced by incubating them for 30 seconds with 500 mM imidazole, a competitive Ni^2+^binding agent. Both these experiments demonstrated that the recombinant cells were specifically bound to the Ni^2+^-NTA beads.

Several reasons could be responsible for the inability of the histidine tags at A2 or Q224 to mediate FimH binding to Ni^2+^-NTA beads, such as presence of the histidine tags interfering with the correct folding of the binding domain of FimH or significantly altering the number of fimbriae on the cell surface. However, we did not quantify the production of transgenic fimbriae nor the amount of transgenic FimH since our primary objective was to identify permissive positions in FimH for histidine tags that would enable robust binding of transgenic cells to their cognate Ni^2+^-NTA agarose substrate. Moreover, in previous studies, binding assays were performed under gentle agitation with cells resuspended in defined M63 salts, unlike ours wherein the cells were resuspended in relatively complex LB media and rotated at 360 degrees. Cells transformed with doubly His-tagged versions of FimH could bind much better than single His-tagged versions at the same residues (i.e. A2 and Q224), suggesting that an extra His-tag improved the accessibility of the binding residues in the modified FimH.

### Binding of *E. coli* via His-tagged FimH activates the same transcriptional cascade as observed with FimH binding to mannose

Using qRT-PCR, the transcriptional response of CSH50(WMHis6) cells bound to Ni^2+^-NTA agarose beads for one hour was compared to that of an unbound fraction of the same cells for a selected set of genes (namely *ahpC, ahpF, dps, grxA, katG, marA* and *ygaP*) that were upregulated consistently and in a temporal manner when CSH50 cells bound to mannose-agarose beads. The qRT-PCR assay of CSH50(WMHis6) cells bound to Ni^2+^-NTA agarose beads revealed that all the genes studied were also upregulated similar to that during wild-type FimH-mediated adhesion of CSH50 cells to a mannose substrate (Table 2). This suggested that at least for the genes investigated, the transcriptomic response of *E. coli* upon attachment to the Ni^+2^-NTA substrate via the genetically modified FimH is similar to the transcriptional response observed in *E. coli* attached to mannose-agarose substrate via wild-type FimH. The aim of this experiment was to identify candidate genes that would be upregulated in a similar fashion irrespective of the FimH ligand and could be used in future as adhesion reporters. Therefore, we did not proceed with checking expression levels of other genes that were differentially regulated upon *E. coli* attachment to mannose-agarose.

### Fimbrial adhesion and biofilm formation- is there a link?

Biofilms are a community of microorganisms attached to a surface and can form on virtually any biotic or abiotic surface.^29^ An integral step in the complex process of biofilm formation is the irreversible attachment to target surfaces and is known to be mediated by adhesive organelles like curli fimbriae and type 1 fimbriae.^30^ A wealth of literature has accumulated about transcriptomic changes associated with biofilm formation, primarily on abiotic surfaces.^31^–^34^

However, relatively few studies have comprehensively analyzed the transcriptional response of *E. coli* following attachment to biotic surfaces. Of note, differential display analysis has been used to demonstrate induction of the iron-starvation response gene, *barA,* upon PapG-mediated fimbrial attachment of a uropathogenic *E. coli* strain to human red blood cells.^11^ Differential display analysis also led to the discovery that the capsular response gene, *kpsD*, is down-regulated upon FimH-mediated fimbrial binding of a different uropathogenic *E. coli* strain to mannose sepharose beads.^12^ Both these studies were performed using pathogenic bacteria and would not necessarily represent changes taking place within benign *E. coli* strains that are the preferred workhorses for synthetic biology applications.

Although the expression pattern of only a small proportion of the genome was perturbed in our current study, there were several interesting parallels with the expression profiles previously observed during biofilm formation. For example, strong induction of OxyR-regulated genes (such as *grxA, dps, katG, ahpCF)* during biofilm growth of asymptomatic *E. coli* in urine, has recently been demonstrated^27^ using microarray-based studies. These authors also reported up-regulation in the multiple antibiotic resistance operon (*marRAB*) in developing biofilms, which is similar to our observations following fimbrial-mediated adhesion. Further, biofilms of *E. coli* strains that are functionally deleted for *grxA* have been shown to have lower survival rates after exposure to metal ions.^35^ Thus, cellular defense against oxidative stress appears to be advantageous for the formation of microbial biofilms.

Our study also identified several genes with unknown functions to be differentially altered. Microarray analysis of biofilm formation has identified *ycfH* to be similarly up-regulated during biofilm formation,^23^ while another, *ycfR*, has been strongly implicated to have a biofilm-related function.^24,36^ Renamed as *BhsA* (for influencing biofilm formation through hydrophobicity and stress response), *ycfR* is presumed to be involved in biofilm formation by participating in indole synthesis.

An important subset of activated genes identified by our study included those with a role in assimilatory sulfur metabolism (i.e. *cysDNC* and *cysJI*). In a similar vein, microarray analyses also revealed that operons involved in sulfate assimilation were induced during biofilm formation,^23,28^ while *E. coli* exposed to a biofilm inhibitor, namely urosolic acid, have shown a consistent repression of genes involved in sulfur metabolism.^37^

While early studies on bacterial biofilms suggested that gene expression within these communities is substantially different from a non-biofilm mode of growth,^38^ recent DNA microarray based studies^24^,^25^,^27^ suggest that biofilms arise due to a unique overall pattern of gene expression instead of a smaller set of biofilm-specific genes. The similarities in gene expression patterns between FimH-mediated adhesion of *E. coli* in this study and biofilm formation in previous studies point to a possible overlap in the machinery activated within cells in these two processes.

To summarize, this study offers a dynamic glimpse of transcriptomic changes triggered upon subjecting *E. coli* to shear stress imposed during fluid flow. Yakovenko et al^6^ conclusively demonstrated that the FimH adhesin forms “catch bonds” with its cognate mannose ligand, leading to shear-enhanced adhesion in which the cells would bind more tightly instead of being washed off when subjected to increased fluid flow or hydrodynamic drag force. Our study was based on the hypothesis that the binding between FimH and its ligand provides a transmembrane mechanical link to transmit forces from extracellular contacts to intracellular structures, resulting in a transcriptional change in the cell. Recent studies have demonstrated that tensile force involved in the catch-bond mediated binding of a recombinant heterodimeric membrane receptor (integrin α^5^β^1^) to fibronectin plays an important role in signaling events mediated by the integrin.^39,40^

We compared the transcriptional responses of attached and unattached fimbriated cells and found that the profile of selected genes investigated was similar whether wild-type FimH was bound to a mannose ligand or modified FimH was bound to a Ni^2+^-NTA ligand. Results obtained using these independently performed experiments gave us enough confidence to believe that the identified genes were indeed altered in response to fimbrial binding and were independent of the particular immobilized substrate. Because we employed two different protocols to dissociate bound cells from the beads (brief vortexing in case of cells attached to mannose-agarose beads versus imidazole displacement for cells attached to Ni^2+^-NTA agarose beads), we believe that the observed transcriptional response was a result of attachment of cells to the beads and was not due to their detachment.

Our study revealed down-regulation of general metabolism and nutrient import accompanied by up-regulation of genes encoding transport systems for antibiotics and drugs, together with induction of a number of genes related to stress responses, specifically those governed by OxyR. A temporal analysis of cells binding to mannose-functionalized surfaces revealed that the transcriptional response was magnified upon prolonged adhesion. We are currently working on further dissecting the response by analyzing the transcriptome at shorter time intervals post-adhesion and functionally validating various genes identified.

Our experiments to engineer recombinant versions of FimH also identified a new permissive position in the FimH sequence (I52) which could be used to insert short peptide sequences without loss of FimH fimbrial assembly and which endowed the transgenic cells with the ability to bind a novel ligand in a complex medium. Similar studies wherein we have engineered another surface protein, OmpA, to bind to novel targets have revealed the presence of a transcriptional response which is distinct from and more pronounced than that observed with FimH-mediated binding (manuscript in preparation). This may partially be explained by the fact that there are only 300–500 fimbriae per cell as compared to over 100,000 copies of OmpA. These results offer exciting possibilities to engineer *E. coli* attachment to different substrates and link it to the expression of proteins of interest.

## Figures and Tables

**Figure 3a.**
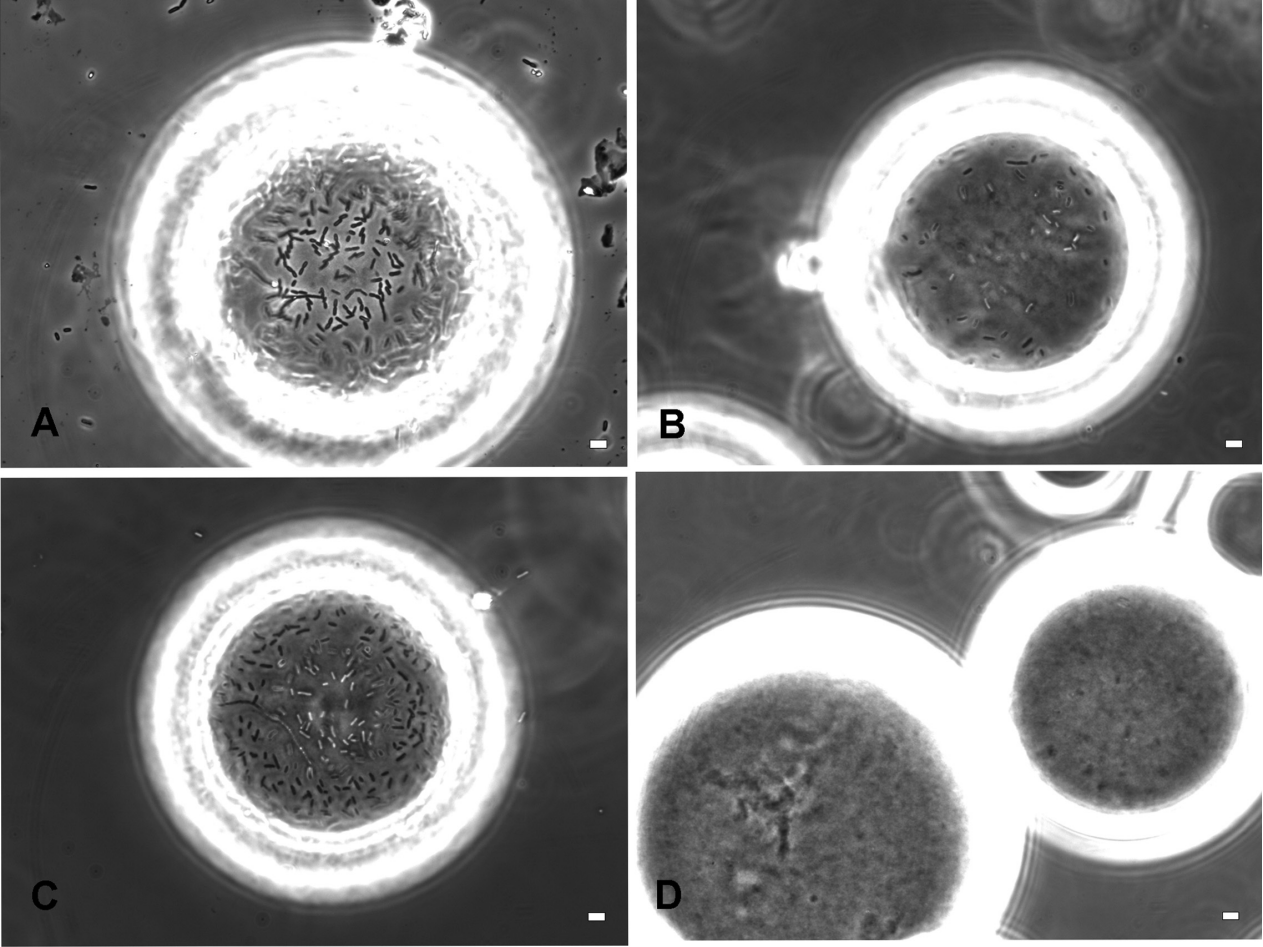
Phase contrast microscopy demonstrating binding of CSH50 cells containing plasmids expressing histidine-tagged versions of FimH. **A)** CSH50 host cells mixed with mannose agarose beads **B)** CSH50(WM2XHis3) and **C)** CSH50(WMHis6) cultured in LBamp50 were mixed with Ni^2+^-NTA agarose beads for 1 h. **D)** same as C) followed by incubation with 0.5 M imidazole for 30 sec. These images are representative of the entire population and a total of at least 100 beads were microscopically observed in 4 independent experiments for calculating the efficiency of binding. The scale bar represents 2 μm.

**Figure 3b.**
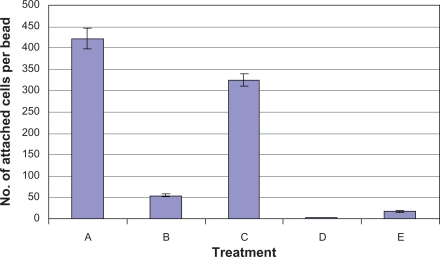
Statistical representation of treatments depicted in figure 3a. Cells bound to mannose/Ni^+2^-NTA agarose beads were counted using the Image Pro Plus software. Treatments: **A)** CSH50 host cells mixed with mannose agarose beads, **B)** CSH50(WM2XHis3) and **C)** CSH50(WMHis6) cultured in LBamp50 were mixed with Ni^2+^-NTA agarose beads for 1 h. **D)** same as C) followed by incubation with 0.5 M imidazole for 30 sec, **E)** CSH50(WM2XHis53) mixed with Ni^2+^-NTA agarose beads. The error bars represent standard deviation.

**Table 1.** Differentially expressed genes (≥1.5-fold) in *E. coli* CSH50 cells attached to mannose agarose beads versus unattached cells.

**Gene^a^**	**Fold-change (*P*-value)**	**Rank**^b^	**Function description**^c^
**I. Protective metabolic pathways**			
**Formaldehyde degradation**			
*frmA*	2.0 (6.6 × 10^−4^)	11	Glutathione dependent formaldehyde dehydrogenase
**Assimilatory sulfate reduction**			
*cysD*	1.7 (3.8 × 10^−4^)	18	1st subunit of sulfate adenyltransferase
*cysN*	1.6 (1.6 × 10^−4^)	26	2st subunit of sulfate adenyltransferase
*cysC*	1.5 (6.8 × 10^−4^)	29	Codes for adenylsulfate kinase
*cysJ*	1.5 (2.2 × 10^−4^)	33	Flavoprotein subunits of sulfite reductase
*cysI*	1.6 (9.9 × 10^−5^)	24	Hemoprotein subunit of sulfite reductase
**Removal of reactive oxygen species**			
*Dps*	2.6 (3.9 × 10^−4^)	6	Protection from multiple stresses including oxidative stress
*grxA*	5.6 (4.9 × 10^−4^)	1	Maintains cytoplasmic reducing environment
*ahpF*	2.6 (9.3 × 10^−8^)	5	Hydroperoxide reductase, H_2_O_2_ scavenging
*marA*	2.0 (3.2 × 10^−6^)	12	Dual transcriptional regulator, multi-antibiotic resistance
*marR*	2.3 (3.2 × 10^−8^)	9	Transcriptional repressor, multi-antibiotic resistance
*katG*	1.5 (8.7 × 10^−3^)	32	Hydroperoxidase, H_2_O_2_ scavening
**Removal of hydrophobic compounds**			
*emrB*	1.5 (2.4 × 10^−4^)	34	Multi-drug efflux protein pump
*emrA*	1.7 (7.2 × 10^−4^)	17	Binds to EmrB, likely to play a role in direct drug transfer via EmrB
*emrR*	1.7 (4.2 × 10^−4^)	19	Transcriptional repressor regulating *emrB* and *emrA*
**II. General metabolism**			
*stpA*	1.8 (6.1 × 10^−5^)	16	H-NS-like DNA-binding with RNA chaperone activity
*fliY*	1.5 (3.8 × 10^−6^)	30	Periplasmic cystine-binding protein
*dcyD*	1.5 (2.7 × 10^−5^)	31	Utilization of cysteine as sulphur source
*ycfR*	1.8 (9.3 × 10^−7^)	15	Indole synthesis
*nirB*	1.7 (7.2 × 10^−4^)	21	Nitrite reductase
*aspA*	0.61 (7.2 × 10^−6^)	−6	Converts aspartate to fumarate and ammonia
*nrdI*	0.64 (2.7 × 10^−4^)	−7	Cofactor for class 1b ribonucleotide reductase
*flgB*	0.53 (5.9 × 10^−8^)	−1	Flagellar body
*fliM*	0.59 (8.8 × 10^−8^)	−4	Flagellar motor
*sdhC*	0.59 (6.2 × 10^−5^)	−3	Succinate dehydrogenase, TCA cycle
*tnaC*	0.57 (3.3 × 10^−6^)	−2	Leader peptide regulating translational attenuation of tryptophanase
**III. Transport-related**			
*marB*	2.4 (2.1 × 10^−4^)	7	Multiple antibiotic resistance protein, putatively exports antibiotics
*glnH*	1.7 (1.2 × 10^−5^)	20	High-affinity glutamine transport
**IV. Not characterized**			
**Putative function**			
*ygaV*	3.5 (1.0 × 10^−3^)	4	DNA binding transcriptional regulator
*ygaP*	4.8 (1.6 × 10^−5^)	2	Membrane protein with hydrolase activity
*yqhD*	2.4 (9.4 × 10^−3^)	8	NADP-dependent aldehyde dehydrogenase
*yeeE*	1.9 (1.5 × 10^−6^)	14	Transport system permease protein
*yqhC*	1.6 (3.4 × 10^−4^)	23	DNA binding transcriptional regulator
*ycfH*	1.5 (1.2 × 10^−5^)	27	Predicted metallodependent hydrolase
*ybaP*	1.5 (9.3 × 10^−7^)	28	Putative ligase
*yicE*	0.61 (3.8 × 10^−6^)	−5	Electrochemical potential-driven transporter
**Function unknown**			
*ygaR*	3.7 (2.5 × 10^−5^)	3	unknown function
*ygaW*	2.1 (7.6 × 10^−5^)	10	Inner membrane protein, unknown function
*ygaM*	1.9 (5.2 × 10^−7^)	13	unknown function
*yeeD*	1.6 (1.2 × 10^−4^)	22	unknown function
*yeeR*	1.6 (1.1 × 10^−3^)	25	Membrane protein, unknown function
*ydiL*	0.66 (1.2 × 10^−8^)	−8	unknown function

a Gene names according to Regulon DB V5.7 database (http://regulondb.ccg.unam.mx/index.html).

b Rank position; 1 = most up-regulated gene in attached cells, −1 = most down-regulated gene in attached cells.

c Function description according to EcoCyc and Cybercell databases, supplemented by specific literature searches.

**Table 2.** Comparison of transcript fold ratios analyzed by qRT-PCR and microarray.

**Gene**	**qRT-PCR (1 h)**	**qRT-PCR (4 h)**	**qRT-PCR (8 h)**	**Microarray (1 h)**	**Microarray (4 h)**	**Microarray (8 h)**	**His-tagged FimH (1 h)**
*ahpC*	1.7^0.002^	4.5^0.001^	4.8^0.002^	1.2^0.2^	2.6^0.5^	3.0^0.5^	n.d.
*ahpF*	1.6^0.002^	4.3^0.002^	5.8^0.005^	2.6^0.4^	3.3^0.18^	8.4^0.5^	8.0^0.002^
*barA*	1.0^0.001^	n.d.	n.d.	1.0^0.01^	0.9^0.01^	0.9^0.03^	n.d.
*Dps*	3.9^0.003^	0.9^0.003^	3.3^0.002^	2.6^0.28^	0.4^0.08^	1.6^0.16^	6.0^0.003^
*grxA*	6.4^0.005^	10.8^0.004^	23.1^0.005^	5.6^0.45^	8.9^0.6^	14.7^1.3^	8.0^0.004^
*katG*	3.3^0.002^	5.0^0.002^	4.0^0.003^	1.5^0.14^	4.7^0.64^	6.0^0.7^	n.d.
*marA*	1.6^0.004^	1.1^0.003^	1.5^0.004^	2.0^0.3^	1.3^0.16^	0.7^0.09^	n.d.
*ygaP*	6.4^0.001^	1.0^0.001^	1.5^0.003^	4.1^0.7^	0.7^0.12^	1.0^0.2^	5.0^0.002^
yqaB*	1.0^0.002^	1.0^0.002^	1.0^0.001^	0.9^0.09^	0.6^0.07^	0.8^0.03^	1.0^0.001^

*qRT-PCR reference gene.

**Abbreviation:** n.d., not determined, the numbers in superscript represent standard deviation. All the data points have P-values less than 0.010.

**Table 3.** Primers used for qRT-PCR.

**Gene**	**Blattner id**	**Primers**
*ahpC*	b0605	5′ - CCTGCTGCGTAAAATCAAAGC
		5′ - GAGACGGAGCCAGAGTTGCT
*ahpF*	b0606	5′ - GAAACCAACGTGAAAGGCGT
		5′ - CTCAGAGAGGCTTTGGCACC
*barA*	b2786	5′ - CGGTGTGCCACGTATGAAGA
		5′ - CCAACAGTTCCAGCAGCTCC
*dps*	b0812	5′ - AAAGAACTGGCTGACCGTTACG
		5′ - GACGCGGCGGTCAGG
*grxA*	b0849	5′ - GTGCGGAAGGGATCACTAAAGA
		5′ - ATAGCCGCCGATATGTTGCT
*katG*	b3942	5′ - TCTAACTCCGTCCTGCGTGC
		5′ - TTCATCACTTTCACCCATGCC
*marA*	b1531	5′ - GGCAGAACGATATGGCTTCG
		5′ - CCTGCATATTGGTCATCCGG
*ygaP*	b2668	5′ - TTAATCGGCGTTGTACTGGGT
		5′ - CAAAAACCGCTGATTCCTGC
*yqaB*	b2690	5′ - AAACCCGCGCCAGACAC
		5′ - GCCGCCTGAATACCGAAAT
